# Supplementary material for: Integrative Analysis of Regulatory Module Reveals Associations of Microgravity with Dysfunctions of Multi-body Systems and Tumorigenesis
Source: Int J Mol Sci. 2020 Oct 14;21(20):7585. doi: 10.3390/ijms21207585 (PMC7589633; doi:10.3390/ijms21207585)
Supplement: Supplementary file 1 [file ijms-21-07585-s001.zip › supplementary_files/supplementary File.docx]

**Integrative analysis of regulatory module reveals associations of microgravity with dysfunctions of multi-body systems and tumorigenesis**


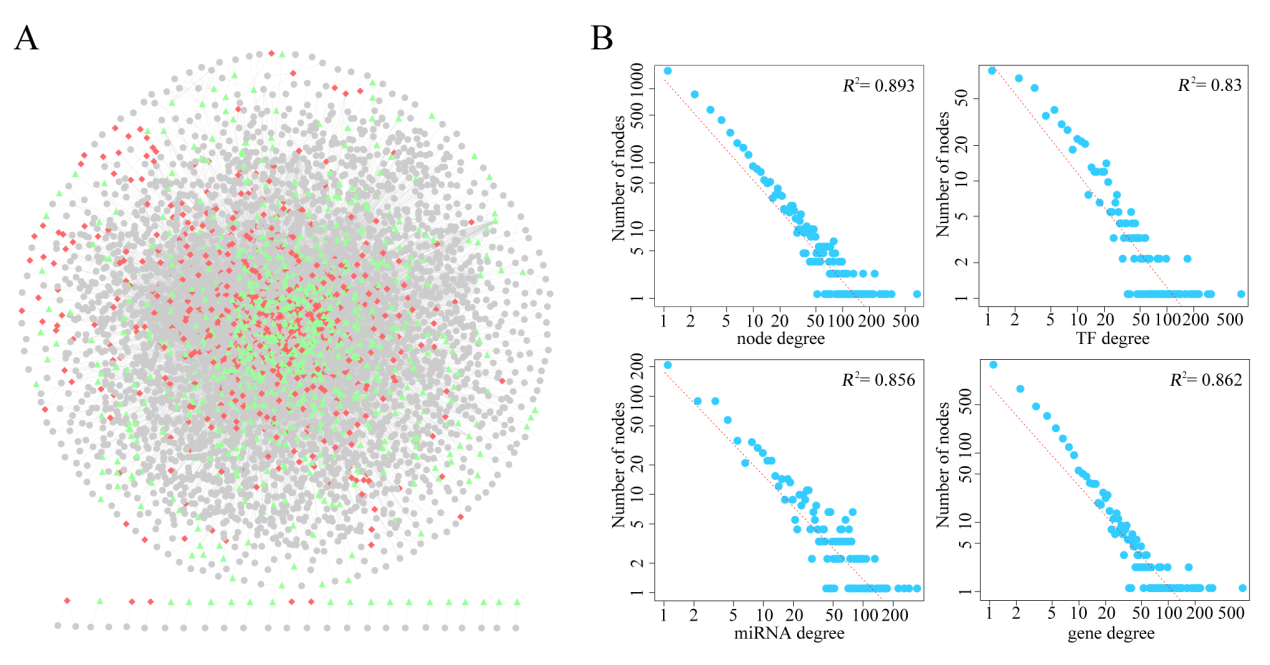


Supplementary Figure 1. TF and miRNA regulatory network and its node degree distribution. A. TF and miRNA regulatory network. Red, green and gray nodes represent TFs, miRNAs and genes, respectively. B. Degree distribution of all nodes, TFs, miRNAs and genes.
